# The potential impacts of human genetics on virus emergence

**DOI:** 10.1073/pnas.2504318122

**Published:** 2025-09-19

**Authors:** Ryan A. Langlois, Jean-Laurent Casanova

**Affiliations:** 1/ Department of Microbiology and Immunology, University of Minnesota, Minneapolis, MN, USA; 2/ University of Minnesota Institute on Infectious Diseases, Minneapolis, MN, USA; 3/ St. Giles Laboratory of Human Genetics of Infectious Diseases, Rockefeller Branch, The Rockefeller University, New York, NY, USA; 4/ Howard Hughes Medical Institute, New York, NY, USA; 5/ Laboratory of Human Genetics of Infectious Diseases, Necker Branch, INSERM, Necker Hospital for Sick Children, Paris, France, EU; 6/ Paris Cité University, Imagine Institute, Paris, France, EU; 7/ Department of Pediatrics, Necker Hospital for Sick Children, Paris, France, EU

## Abstract

Human monogenic traits can confer resistance to viral infection in exposed individuals or predisposition to severe disease in infected individuals. Enhanced susceptibility can be driven directly by mutations in genes essential for control of the virus or indirectly via the production of auto-antibodies against components of host defense. While the impact of viruses on individuals carrying these genotypes permitted their identification and has been amply studied, little is known about the impact of these human genotypes on the natural history of viruses, including not only persisting but also emerging viruses. We envisage several scenarios, including the possibility that genetically susceptible individuals serve as patient zeros, super-spreaders, or mutation incubators, or that genetically resistant individuals even permit the selection of new viral mutants. Viruses are continually shared between individuals and even host species, where they can benefit from adaption to new environments. Current human viruses, as well as novel viruses from animal reservoirs, will continue to threaten the human population. Improvements in the scale of human genomic sequencing and analysis will permit testing hypotheses about the impact of human genetics on the origin and trajectory of viral infections, including future pandemics, which may ultimately help to prevent or curtail impending outbreaks.

## Introduction

In human populations, there is vast interindividual variability in the control and outcome of any viral infection. Human genetics can explain unusual resistance or vulnerability to disease in patients with multiple or even specific viral infections(1, 2). A forward genetic approach has uncovered two cases of monogenic resistance to viral infection, with autosomal recessive CCR5 and FUT2 deficiencies conferring resistance to HIV and norovirus, respectively(3–5). This approach has also uncovered genetic predispositions to severe outcomes with what are more commonly benign and well controlled, even often silent, viral infections, in otherwise healthy individuals normally resistant to other viruses. An increasing focus on human genetics of severe viral diseases has demonstrated that single-gene inborn errors of immunity (IEI), monogenic mutations that impair components of intrinsic (herein defined as operated by cells other than leukocytes), innate (operated by leukocytes other than T and B lymphocytes), or adaptive immunity (operated by T and B lymphocytes), can be causal for a growing number of viral diseases, albeit typically in only a small minority of cases(6, 7). Advances in sequencing have greatly facilitated the forward human genetic analysis of determinants of infection outcome in patients suffering from severe viral diseases or of individuals resistant to viral infection. The pace of discovery of new IEI underlying viral diseases studied genetically is increasing, and it is also probable that other types of viral diseases will be found to be caused by new IEI. There are also probably other examples of genetic resistance to viral infection yet to be discovered and characterized, as illustrated with resistance to parvovirus(8).

Approximately 1,000 viruses are known to infect humans and there are many more with human-tropic potential(9). Over the last century, there have been several significant occurrences of viral zoonosis including 1918 influenza, HIV, Ebola, Zika, and SARS-CoV-2 among others. Humans are constantly exposed to new viruses, and this will probably increase due to climate change, land use change, expanding animal trade, and increasing human travel and population(10). The trend is particularly clear for arboviruses. Moreover, the areas of endemicity for known viral pathogens tend to increase steadily, as neatly illustrated by West Nile virus(11, 12). Viruses that are on the brink of crossing species barriers to emerge in the human population face two immediate concomitant obstacles. First, viruses need to exploit host factors to enter cells and complete their life cycle. Second, and prior to any intervention of leukocytes in their innate and adaptive compartments, viruses must evade multiple layers of cell-intrinsic immunity, which can operate in any of the > 500 known cell types of the human body. This can be driven by type I interferons (IFN-I) through the downstream effector proteins induced. It is possible that novel viruses may place new selective pressure on the human population revealing new IEI. For example, why were some IEI revealed by SARS-CoV-2 but not by influenza or seasonal CoVs(1)? It is hard, if not impossible, to model the role of interindividual variability in animal studies. Model animals are not human, with no history of co-evolution with human-tropic viruses and most animal models, mice in particular, were developed to be inbred, precisely to erase inter-individual genetic diversity. Further, they are typically housed in unnatural, specific pathogen-free conditions to control for the confounding variable of previous infections(13). This places a greater impetus on evaluating mechanisms of genetic disease susceptibility/resistance within human populations to help identify patients who may be particularly susceptible to emerging virus, develop novel therapies, and surveillance strategies to prevent and contain new pandemics(14).

**Figure 1.**
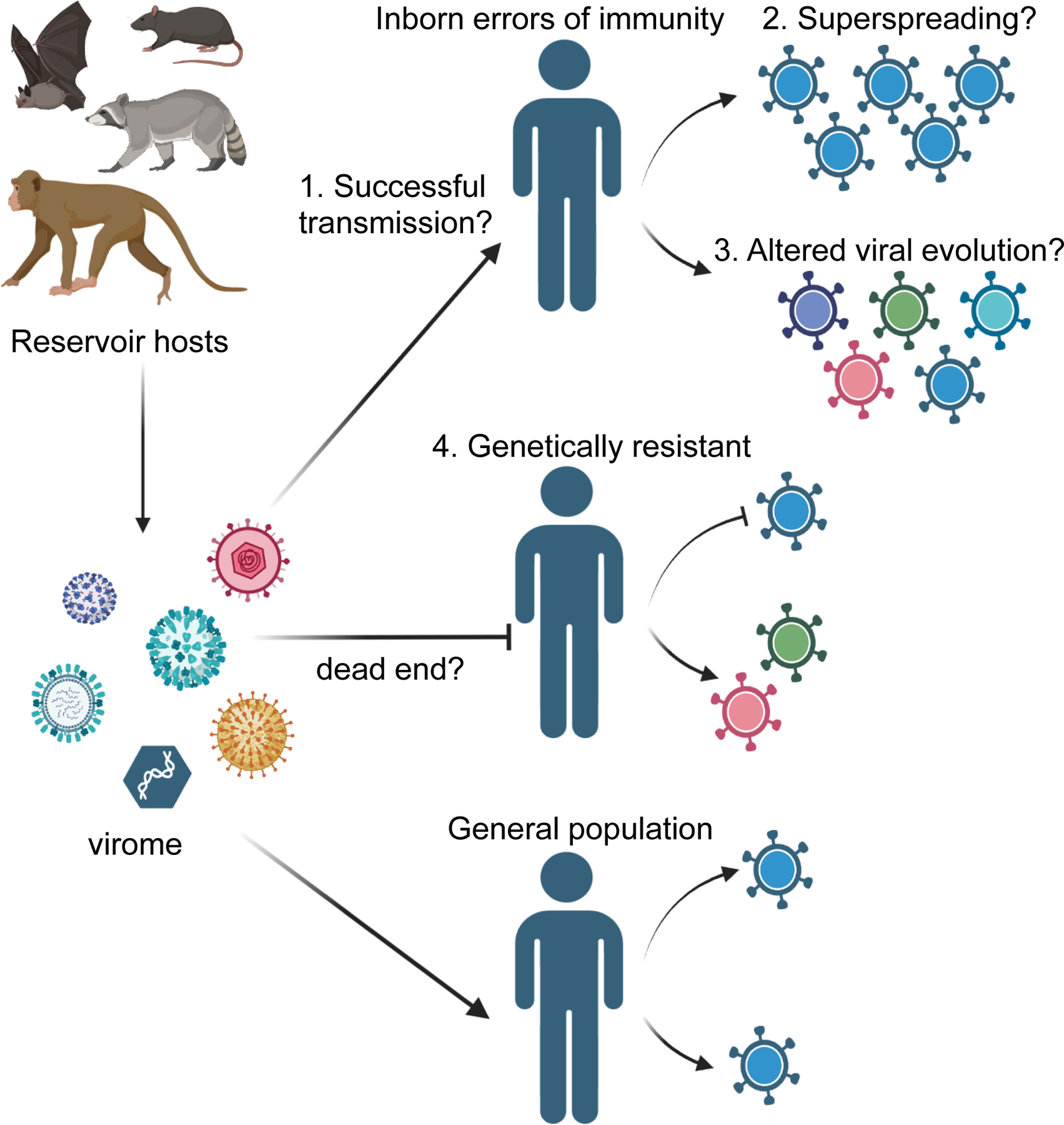
Humans are constantly exposed to viruses from the global virome. Human genetics can have an array of potential impacts on virus emergence. Patients with inborn errors of immunity may be index cases for new infections (1), spread virus more efficiently (2), or alter virus evolution trajectories (3). Patients may have mutations that make them genetically resistant to virus infections (4), with unknown consequences for virus evolution.

IEI and their autoimmune phenocopies have been amply reviewed from multiple angles, particularly focusing on the patients being targeted by, or victims of viruses. Yet, to our knowledge, their potential contribution to the emergence, spread, or evolution of new viruses has not been discussed. How viruses impact humans with monogenic susceptibility or resistance, is well understood, but what these individuals can do to the corresponding viruses and how they may shape their fate, particularly for emerging viruses, is unknown. For novel viruses entering the human population there will be little or no preexisting adaptive immunity, placing a larger burden on intrinsic (typically non-leukocytic) and innate (typically leukocytic) immunity for control. Patients with IFN-I IEI or autoantibodies neutralizing IFN-I (IFN-I autoAbs), or deficient in other components of intrinsic or innate immunity may be particularly susceptible. For most endemic viruses, primary infections occur early in life. Emerging infections differ and can occur across all age groups. We do not consider maternally and sexually transmitted viruses, which have their own specific patterns of transmission. Here we discuss how human variation in immunity, genetic or otherwise, including monogenic genotypes that confer resistance to viruses among exposed individuals or predisposition to severe disease among infected individuals, may impact the biology of viruses as they emerge or persist in humans (Figure 1).

## Innate immunity as a barrier to cross species infections

**Figure 2.**
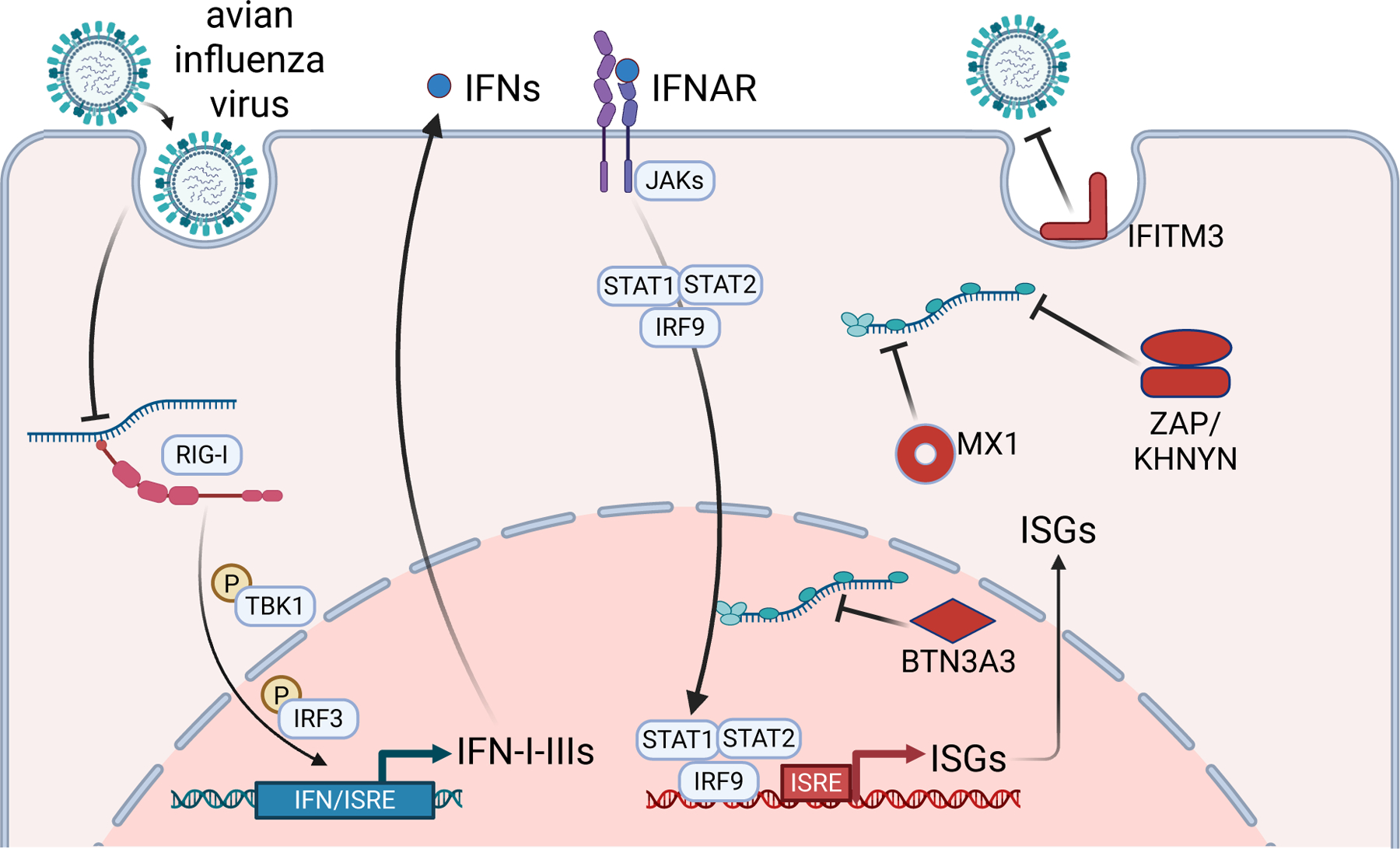
Innate immune barriers to influenza virus infections. Left side: antiviral sensing, production of IFN-I and induction of antiviral genes after influenza infection. IEIs have been identified in all components of this signaling pathway (Table 1). Right side: Interferon stimulated genes that restrict cross-species infection with avian influenza.

The innate immune system presents a potent barrier to cross species infections (Figure 2). A hallmark of antiviral immunity in jawed vertebrates is the IFN-I response. Viral intermediates or by-products of replication are detected, leading to the production of anti-viral molecules, including type I and III IFNs. IFNs then lead to the production of hundreds of genes, most of which are thought or shown to be antiviral (and others regulators), which make neighboring cells inhospitable to virus infection and replication. Intrinsic and innate immune genes are under selective pressure from microbes and are heavily divergent between species(15–18). Given that viruses are often adapted to a particular host species, this poses a hurdle for successful cross-species jumps. Therefore, novel viruses which have evolved to antagonize the IFN activity of the reservoir host, may be only poorly able to subvert intrinsic immunity in humans.

Influenza provides a test case for how IFN-I mediated immunity can provide a barrier to cross-species infections (Figure 2). Species-specific restriction of influenza by MXA can pose a strong infection barrier. MXA potently restricts influenza in mammals but not avian species (19, 20). Therefore, avian influenza viruses have not evolved MXA evasion mechanisms and are restricted in mammals. Variants in *MXA* that predispose humans to severe infection with avian influenza viruses provide an opening for cross species infections (21). We identified an additional restriction mechanism against influenza in mammals through ZAP and KHNYN which restrict by targeting viral RNA. ZAP is nonfunctional in avian species and KHNYN is completely absent in birds, presenting a barrier to zoonosis (22). Similarly, IFITM3 restricts cross-species influenza infections with potential loss of function alleles present in the human population (23, 24). Further, IFITM3 deficiency in mammals reduces dose needed for avian influenza infection and led to enhanced host viral adaptation (24). Finally, the primate specific ISG BTN3A3 which targets RNA replication can prevent infection with avian influenza viruses (25). Together these data demonstrate how antiviral genes can pose a barrier to cross-species infections. Another classic example of innate immunity posing a cross-species barrier is APOBEC3G restriction of SIV infections in humans and virus adaptations in VIF to target APOBEC3G to successfully establish in the new host (26, 27). Despite exposure to SIV revealed from serological studies, HIV-1 only emerged in humans 4 independent times(28–30). It is interesting to speculate that the first humans infected with HIV had reduced barrier immunity with potential mutations in restriction factors (e.g. APOBEC3G, SAMHD1, TRIM5a, tetherin) or antiviral IFN pathways, permitting the virus to gain a foothold and eventually the mutations needed to competently spread within the new species. Innate immunity as a barrier has been extensively demonstrated in reverse zoonosis studies with human viruses experimentally inoculated to mice where innate immune genes/products frequently need to be manipulated to establish infection. For example, IFN signaling needs to be disrupted in mice either genetically or through IFNAR blocking antibodies for successful infection with many flaviviruses including Zika, Dengue, and Yellow Fever (31–33). Together, these data suggest that intrinsic and innate immunity pose a significant potential barrier to emerging virus infections, more so than adaptive immunity.

## Intrinsic and innate inborn errors of immunity

Human type I IFN immunity is essential for the early control and prevention of several viral infections(34). This has been revealed through human genetics in patients with severe viral disease where defects have been found across the pathway from virus sensing through type I IFN signaling (Table 1). Observations *in natura* have demonstrated that antiviral sensing (e.g. TLR3, TLR7, MDA5) is critical for preventing severe disease during viral infections, and may also reduce the innate immune barrier to cross species infections(35–38). There are also patients with mutations in genes that impact IFN-I signaling. Bi-allelic mutations in the IFNAR1 or IFNAR2 chain of IFN-I heterodimeric receptors are the prototypical type I interferon (IFN-I) IEI. While globally rare, recessive complete defects of IFNAR1 and IFNAR2 are due to common alleles in Western Polynesia and the Arctic, respectively (39, 40). Moreover, a dominant form of IFNAR1 deficiency involves an allele that is common in South China (41). Downstream of IFN-I receptor mutations in TYK2 and STAT1 or STAT2 can also lead to defects in IFN-I responses. All these patients suffer from increased disease with a range of virus infections (34, 42). As discussed above there are some variants in IFN stimulated genes but fewer of these have been associated with severe viral diseases(21). This may be due to redundancy in antiviral genes and because ISGs can be specific to viruses/virus families making them harder to identify and requiring larger patient cohorts.

In addition to genetic lesions in immune genes, autoantibodies (autoAbs) which neutralize cytokines can phenocopy IEIs. For example, autoAbs against IFN-I phenocopy loss of function mutations in IFN-I signaling pathway, and substantially suppress this intrinsic antiviral defense system. These IFN-I autoAbs are much more common globally and strikingly can explain 5–40% of severe disease for a growing range of emerging/reemerging viral infections, particularly pulmonary and cerebral infections(34). These autoAbs are found in about 0.5% and 5% of healthy individuals under and above 70 years old, respectively(43, 44). While the mechanisms driving induction of IFN autoAbs are not understood in the elderly, they can result from inborn errors of tolerance in children and younger adults, thus adding another significant monogenic cause of viral disease susceptibility(45). Moreover, autoAbs against IL-27, like inherited deficits of the IL-27 receptor, can underlie severe infection with Epstein-Barr virus(46). In addition to IEI and their autoimmune phenocopies, there can be deficiencies that are acquired throughout life (e.g. HIV/AIDS and immunosuppressive drugs). Combined, these immunodeficiencies place affected humans at risk for severe disease with both known and novel viruses.

## Are people with IEI/IFNautoAbs potentially patient zeros?

Humans are constantly exposed to viruses from zoonotic reservoirs. Serosurveillance studies have identified people with reactivity to novel and emerging viruses from animal hosts(47–51) Strikingly, these analyses have demonstrated tens of thousands of potential novel SARS-like infections per year in China(52). A caveat to these studies is that the level of virus replication and antigen production required for seroconversion is likely very high and therefore serological assays may not capture many cross-species dead-end infections as a virus is sampling new hosts. Fortunately, while exposure rates can be high, zoonotic viruses rarely establish themselves in the human population. People with IEI impacting intrinsic or innate immunity may make an ideal initial host (patient zero), as a major barrier of host defense would be compromised. This would provide viruses the chance to gain a foothold in the human population where they would otherwise be thwarted. At first glance it may seem unlikely given the *perceived* rare frequency of IEI and exposure to novel viruses. However, as discussed above IEI and IFN autoAbs combined are not as rare as they were thought to be only 20 years ago. A single patient with a novel virus infection may allow sufficient viral adaptation for transmission to other humans, as dead-end cross-species events are most common in people without these immunodeficiencies. Immunocompromised transplant patients with significant exposure to wildlife and agricultural animals present with infectious diseases that are rarely found in the general population(53). Reservoir and host virus screening and sampling coupled with phylogenetics can help to identify potential origins of new pandemics(28, 54–57). However, it is still difficult, if not impossible, to identify index patients during novel outbreaks. Therefore, patients harboring IEI serving as a facilitator to virus emergence is speculative but there is evidence to suggest this may be possible. While there is currently no strong evidence on the biology or circumstances of patient zeros, it is tempting to speculate that viral emergence could occur in patients with severe lesions in IFN pathways, or unknown genetic defects, in whom a decrease in initial defenses could permit viral replication and adaptation to novel host machinery.

## Do patients with IEI disproportionately contribute to virus spread?

In addition to heterogeneity in the outcome of viral infections, there is also significant interindividual variability in the transmission of viruses. The basic reproductive number (R0) represents the number of new infections seeded by any individual amongst a susceptible population. Viewing viral pathogenesis and spread at population level has obfuscated the genetic cause of individual disease and can mask individual causes underlying the disparities in virus transmission rates. By assessing virus spread at the individual level, it has been revealed that a disproportionate number of cases are transmitted by few individuals. This phenomenon has been termed “Super-spreading” and has been observed for transmission of SARS-CoV-1 and −2, MERS, measles, and Ebola(58–62). There are obvious social and behavioral candidate causes for super-spreading, but potential host biological causes, to our knowledge, have not been immunologically or genetically evaluated. Patients also demonstrate significant heterogeneity in the level of shedding of many viruses including SARS-CoV-2 and influenza virus and the biological mechanisms are yet undefined(63, 64). Importantly, evaluation of viral genomes from super-spreading events do not show virus strain or sequence differences, suggesting that the virus is not the culprit, further implicating the host(65).

We postulate that IEI or their autoimmune phenocopies, including those affecting I-IFN, may play a role during virus transmission. Even if people with IEI are not patient zeros, or the match that starts a fire, they may still serve as a fire accelerant. Patients with IEI may be key individuals early in a pandemic. The human genetic and immunological basis of silent viral spread has not been studied but should be. This could reveal new types of IEI, with little individual costs but greater populational cost. For example, patients with agammaglobulinemia are highly vulnerable to enteroviral encephalitis, but often only long after having chronically shed enteroviruses in their feces, thereby transmitting the virus far more than others(66). Of course, clinically overt infections can also contribute to viral spread – albeit clinical manifestations by themselves tend to prevent viral spread via bed-ridding the patient and alerting the contacts. The contagion is greater when people are silently infected, as they may still pursue social interactions, potentially providing a genetic explanation for the known social causes of super-spreading. If patients do not have life-threatening disease they may not come into the clinic and may not be evaluated genetically. Human genetic variants could also potentially result in tolerance, where pathology is reduced but replication and potentially shedding remain high(67).

Patients with IEI might also shed virus at higher loads and/or for longer periods of time (discussed more below) increasing the window in which they could transmit virus. IEIs may result in such severe disease that death or hospitalization occurs prior to the ability to widely spread the infection. In contrast, mutations that confer tolerance or impact adaptive immune responses may result in prolonged shedding increased transmission potential, with an impact at the population level but not on the fitness of the carrier. Fortunately, this is a testable hypothesis. “Super-spreading” individuals have been identified during previous outbreaks(65), and could be evaluated genetically to identify potential mutations associated with transmission. Interestingly, older males disproportionally contribute to virus spread via “super-spreading” and have a higher prevalence of IFN autoAbs(68). This too could be tested, and a correlation would suggest the potential role for IFN autoAbs in virus shedding and transmission. Finally, while individuals carrying a dominant-negative IFNAR1 allele might be patient zeros, they might also perhaps be super-spreaders(41). If genetic and mechanistic causes of “super-spreading” are identified, then patients more likely to spread viruses could take increased precautions and use non-pharmaceutical interventions (e.g. masking) to help blunt the spread of new epidemics and pandemics.

## Do IEI contribute to viral evolution within the new human host?

Human genomes impart pressure on viral populations resulting in adaptations to evade intrinsic, innate, and adaptive immunity. Patients with intact intrinsic and innate immunity may fully suppress emerging virus infections, preventing intra-host virus adaptation. Therefore, the specific lesion in immunity may have a significant impact on capacity a virus to adapt to a new host. Additionally, virally infected patients with IEI, or who have acquired immunodeficiencies, may display longer virus shedding, indicating a prolonged time between infection and viral clearance. This can lead to increased intra-host viral mutation accumulation and have a significant impact on viral evolution. Adaptive immune defects from IEI, HIV, bone marrow transplants, among others have led to prolonged shedding and increased generation of virus variants with oral polio vaccine, influenza, parainfluenza, and SARS-CoV-2(69–74). There is some evidence to suggest that the SARS-CoV-2 variants generated in immunocompromised patients could be the mutational sources for new strains which rapidly emerged after widespread immunity from vaccination and natural infection(75, 76). Similarly, there is data to suggest that patients who are immunosuppressed may serve as sources for variants that drive new norovirus pandemics(77).

It might be viewed as surprising that there are no studies demonstrating intrinsic IEI or IFN autoAb leading to altered virus evolution. Patients with IFN autoAb have been demonstrated to have increased virus loads and longer shedding (78–80), both conditions that can favor generation of variants. To our knowledge viruses have not been sequenced from these patients. Residual levels of IFN-I signaling in IFN autoAb patients might provide partial pressure, enhancing generation of IFN-I antagonist mutants. Additionally other intrinsic IEI are probably not complete defects in intrinsic immunity. For example, people with complete IFNAR1 or IFNAR2 deficiency still have type III IFN activity. Likewise, IRF7-deficient patients, with a near complete deficiency of type I and III IFN induction, with the exception of IFN-beta(38, 81), can still activate antiviral genes through IRF3, providing partial pressure for type I IFN escape mutants to arise. Animal studies have demonstrated that the absence of IFN pressure results in altered virus mutation trajectories and dissemination(82–84). Replication in new tissues and cell types may present new pressures with unpredictable viral evolutionary trajectories that may impact replication success in new immunocompetent or deficient hosts. Intrinsic IEI may select for viruses with enhanced replication capacity. Larger virus loads and decreased shedding bottlenecks may in turn increase fitness in a new immunocompetent host. Virus diversity is essential to overcome the diverse arsenal presented by intrinsic innate immune responses. While many mutations in intrinsic immunocompromised hosts are probably driven by purifying selection, emerging viruses coming from different host species are under different selective forces and relaxing immune pressures may result in mutations that would be deleterious in the reservoir but adventitious within a new host.

Viruses can also rapidly evolve through recombination or reassortment. Coinfection with different strains of the same virus can lead to virus recombination or reassortment resulting in access to more sequence space and creation of new virus genotypes. These coinfections can result in leaps in virus evolution that may not be possible with single base pair changes from low fidelity replication. Recombination and reassortment are responsible for the origins of many human viruses including pandemic influenza viruses, HIV, western equine encephalitis virus, and SARS coronavirus (27, 85–87). Type I IFN responses can also help to curtail coinfections. Infection with the first virus strain triggers an IFN response preventing infection with a second virus strain(88). Patients with intrinsic IEI or IFNauto antibodies may serve as mixing vessels, supporting coinfections leading to increased rates of viral recombination or reassortment. For example, someone with IFN autoAbs with a seasonal influenza could encounter animals with avian influenza, leading to the productions of new influenza strain not possible in immunocompetent hosts.

New variants of zoonotic viruses generated in IEI patients may contribute to more rapid evolution to human hosts, providing a larger viral mutant population for subsequent selection. IEI may also permit viruses to transmit with a looser genetic bottleneck, allowing seeding of more variants during transmission and speeding the process of virus evolution. These hosts would serve as mutation incubators, not so much selecting for viral variants as permitting their emergence via sheer numbers and diversity. Recently a genome-to-genome approach, which uses the genomes of both virus and host, has been able to identify impacts of human genetics on virus evolution. These studies have revealed human variants which shape the evolution of HCV, HBV, EBV, and HIV(89–94). For example, patients with a nonfunctional IFNL4 gene results in increased virus burden and mutations(89, 94). Together, these studies demonstrate that human genetic variation can have consequences for the virus. Evolutionary distance between the reservoir species and humans may also shape virus evolution. Viruses emerging from phylogenetically closer reservoirs may be more primed to take advantage of a novel host. In such a scenario, IEI may not be as impactful, as these viruses may have already developed innate or intrinsic immunity evasion mechanisms that would be successful in humans. However, viruses emerging from reservoirs at larger evolutionary distances may need the foothold created by IEI.

## Do human monogenic resistance to viruses shape virus evolution?

Another human genotype might also contribute to emerging virus evolution and spread: the resistors, or hosts with cells that strongly or completely prevent replication. Instead of serving as a mutation incubator, a resistor host would serve as a selective filter. The cells of the resistant individual might select for mutant viruses that find alternative routes of entry and replication. Within the population of viruses these hosts would be exposed to, rare viral mutants may bypass the resistant human genotype and proceed to full replication. In turn these rare typically inviable mutants could propagate and spread to other individuals. This would potentially give the virus new paths to infections in humans and in the long term may pose more threats to the population.

There is some evidence for this principle, and as with many discoveries in genetics, plant geneticists were pioneers in this area. Polymorphic regions in plants can provide resistance to infections and resistant individuals can select for virus mutations that make viruses better generalists, allowing infections of more genotypes(95). Could this phenomenon occur in humans? Resistance has also been observed in humans with clear mechanisms for two viruses.

CCR5-negative patients may provide an opportunity for an HIV mutant to find another new route of infection. However, this has not been observed in patients who were HIV-positive and received hematopoietic stem cell transplants from CCR5 negative patients(96). Similarly, mutations in FUT2 protect against norovirus and rotavirus infections yet it may be possible for the virus to find alternative entry pathways, particularly if the resistance is not absolute(4, 5). Incomplete resistance may lead to substantial virus evolution as occurs in the face of antiviral drug pressure. Mutations in an HCV receptor SCARB1 result in reduced HCV loads demonstrating incomplete entry resistance and potentially allowing a path to escape use of this host factor(97). Surprisingly only two examples of *bona fide* monogenic resistance to viruses in humans are known. This is paradoxical, as epidemiological studies show that there is a plateau of seropositivity for almost every virus studied. Not everyone is Epstein Barr virus, cytomegalovirus, or herpes simplex virus seropositive, despite increasing social contacts and mass travel in recent decades. Identifying resistors is complicated by that fact that it is difficult to prove that an individual was never truly exposed to a virus. Seronegativity and lack of detectable virus-specific T cells may indicate lack of exposure, lack of infection, or eradication after infection by intrinsic/innate immunity only and prior to adaptive immune activation(98). Serology tests can suffer from sensitivity issues, as exemplified by not all SARS-CoV-2-positive patients seroconverting(99, 100). Additionally, both antibody- and T cell activation-based assays to measure prior exposure suffer from potential cross reactivity to related pathogens. Large scale surveillance studies and new virus detection and exposure methods will be needed to be overcome these barriers to identify and study rare novel resistance mechanisms in humans.

While not yet well characterized in nature, it is also possible that polymorphisms in IFN-stimulated or restriction factor genes could enhance control of new infections. Antiviral immune effectors evolved to fight past infections and are under selection. Some antiviral genes or regions of these genes may accumulate neutral mutations through genetic drift which may now be advantageous against a new emerging virus. Because these mutations arose through genetic drift, they may not appear independently in other species making comparative genomics predictions for improved function difficult. Additionally, genotypes that are neutral against endemic infections may have advantageous or deleterious phenotypes when challenged by a novel virus. There is some experimental evidence for improved antiviral gene function through mutation. For example, experimental mutations in MXA and APOBEC3 can dramatically improve their antiviral action(101, 102). It has been well established across many host species, from plants to animals, that there is an inverse correlation between genetic diversity and infectious disease susceptibility(103–105). Interestingly, Likewise, some human populations have lost some genetic diversity during bottlenecking events(106). It is interesting to speculate that this has made us more susceptible to infections over time because there were fewer mutations to select from. Can infection of patients with resistance phenotypes drive new evolutionary trajectories of viruses? For example, could norovirus or HIV overcome the resistance barriers imposed by CCR5 and Fut2 mutations? Could novel resistance mechanisms drive new or more efficacious antiviral antagonism? There may also be other undiscovered resistance mechanisms that provide only partial protection. Interestingly two viruses which infect related primate species and cause disease, foamy viruses and arteriviruses, have not crossed the species barrier into humans. Humans probably have viral resistance mechanisms yet to be discovered. Patients with mutations in these unknown genes may allow for these viruses to replicate in humans and expand their species tropism. This is a branch of genetics of infectious diseases that deserves more study.

## Concluding remarks

The role of host genetics underlying severe disease after exposure to microbes has been evident since the early 20^th^ century thanks to various classical genetic studies, ranging from linking host genetics with fungal disease in plants, to connecting host genetics with tuberculosis in humans(2). With the advent of molecular genetics, the impact of monogenic genotypes as the cause of resistance to viral infection among exposed individuals or severe diseases among infected individuals has only been broadly appreciated more recently(1, 107). It has been well established that immunosuppressed people are more susceptible to novel virus infections. For example, patients with acquired immunodeficiencies and IEI targeting adaptive immunity have expanded viromes including presence of novel viruses(108, 109). However, we are not aware of any in-depth virome characterization in patients with type I IFN IEI or IFN autoAbs, or even any IEI of innate or intrinsic immunity. To date, the impact of IEI on emergence, propagation, and evolution of new viruses into the human population has not been considered, but we argue it clearly should be. Here we provide a conceptual framework of how monogenic lesions may break down the barrier to cross-species infection, increase transmission, and drive virus evolution and adaption, providing testable hypotheses for future outbreaks. Monogenic resistance to infection can be viewed like a dam that can break, potentially leading to an influx of new viruses into humans. It is important to note that patient cases of novel and emerging viruses can be stigmatizing to individuals and populations, and often the stigmatized population is not even the original source of the contagion (e.g. “Spanish flu” which was circulating prior to reports of the infection from Spain(110)). If human genetics does impact the emergence or persistence of viruses, it will be imperative to prevent any stigmatization of affected individuals and populations; these findings would be medically helpful, for the patients and their populations.

First proposed by JBS Haldane in 1949, the impact of microbes on the genomic evolution of all host species through selection is now widely recognized(111). The mutational scars from past pandemics litter our genome(112). Integration of virus genetic elements have radically shaped our evolutionary trajectory (e.g. placental formation from viral syncytia forming proteins) and can act as restriction factors for new infections (e.g. Fv1 in mice)(113, 114). New viruses entering the human population can have unpredictable consequences via a multiplicity of virus-host interactions. Emerging infections with high mortality can select for resistance alleles, shaping human genomes over time. Mutations that predispose individuals to severe disease might not be rare. Fortunately, modern medicine can largely prevent an otherwise fatal infection, permitting these susceptible genotypes to persist and even spread, which may increase their impact on emerging infections in the future. In contrast, the impact of the host on the evolution of viruses has received less attention, even though these microbes evolve much faster. It is possible that control of novel viruses will be dependent on unappreciated antiviral factors that have not been under selection. This would then provide new selection pressures to the viruses altering their evolutionary trajectories in unpredictable ways.

## Figures and Tables

**Table 1.** Innate inborn errors of immunity impacting virus infections

Gene	Function	Human phenotype	References
TLRs 3, 7, 8, 9	Microbial detection	HSV-1 encephalitis, severe viral infections	(115)
UNC93B1	Signaling chaperone	HSV-1 encephalitis, severe viral infections	(116)
RIGI	Viral RNA detection	Severe influenza infections	(117)
MDA5	Antiviral sensing signal transduction	Severe viral infections	(118)
TBK1	Antiviral sensing signal transduction	HSV-1 encephalitis	(119)
TRIF	TLR signal transduction	HSV-1 encephalitis	(120)
TRAF3	Antiviral sensing signal transduction	HSV-1 encephalitis	(121)
IRF3	Antiviral sensing transcription factor	HSV-1 encephalitis	(122)
IRF7	Antiviral sensing transcription factor	Severe influenza infections	(38)
IFNAR1, 2	Interferon receptors	Severe viral infections	(39–41)
TYK2	Interferon signaling	Severe viral infections	(123, 124)
STAT1, 2	Interferon signaling transcription factors	Severe viral infections	(125, 126)
IRF9	Interferon signaling transcription factor	Severe influenza infections	(127)
OAS L, 1, 2,3	Viral RNA sensing and degradation	Severe viral infections	(128)
MXA	Blocks virus replication	Increased influenza infections	(21)
IFITM3	Blocks virus entry	Increased influenza infections	(23)
